# Improving chemotherapeutic efficiency in acute myeloid leukemia treatments by chemically synthesized peptide interfering with CXCR4/CXCL12 axis

**DOI:** 10.1038/srep16228

**Published:** 2015-11-05

**Authors:** Xiaojin Li, Hua Guo, Hongyang Duan, Yanlian Yang, Jie Meng, Jian Liu, Chen Wang, Haiyan Xu

**Affiliations:** 1 Institute of Basic Medical Sciences, Chinese Academy of Medical Sciences & Peking Union Medical College, Beijing 100005, P. R. China; 2 National Center for Nanoscience and Technology, Beijing 100190, P. R. China

## Abstract

Bone marrow stroma can protect acute myeloid leukemia (AML) cells against chemotherapeutic agents and provide anti-apoptosis and chemoresistance signals through secreting chemokine CXCL12 to activate its receptor CXCR4 on AML cells, resulting in minimal residual leukemia and relapse. Therefore disrupting the CXCR4/CXCL12 axis with antagonists is of great significance for improving chemosensitivity and decreasing relapse rate. In a previous study, we reported a novel synthetic peptide E5 with its remarkable effect on inhibiting CXCR4/CXCL12-mediated adhesion and migration of AML cells. Here we presented E5’s capacity of enhancing the therapeutic efficiency of various chemotherapeutics on AML *in vitro* and *in vivo*. Results showed that E5 can diminish bone marrow stromal cell-provided protection to leukemia cells, significantly increasing the apoptosis induced by various chemotherapeutics in multiple AML cell lines. In an AML mouse xenograft model, E5 induced 1.84-fold increase of circulating AML cells out of protective stroma niche. Combined with vincristine or cyclophosphamide, E5 inhibited infiltration of AML cells into bone marrow, liver and spleen, as well as prolonged the lifespan of AML mice compared with mice treated with chemotherapy alone. In addition, E5 presented no toxicity *in vivo* according to the histological analysis and routine clinical parameters of serum analysis.

Poor prognosis of acute myeloid leukemia (AML) with current treatments is mainly due to the relapse of the disease post chemotherapy^1^,^2^. In the last decade, an emerging concept has proposed that interactions between leukemia cells and the bone marrow microenvironment are the major cause of the leukemia relapse^3^,^4^,^5^,^6^. In the cross-talking between them, leukemia cells highly expressing the chemokine receptor 4 (CXCR4) are recruited into the bone marrow by stromal cells constantly secreting the chemokine ligand 12 (CXCL12)^2^,^7^,^8^. Leukemia cells that adhere to the stromal cells can reside in the bone marrow to acquire anti-apoptosis signals and favorable conditions for survival and growth^9^,^10^,^11^,^12^. Thus through CXCR4/CXCL12 axis leukemia cells are protected by the stromal cells from cytotoxic chemotherapeutics and represent a reservoir for minimal residual disease and relapses^2^,^13^,^14^.

Given the central role of the CXCR4/CXCL12 axis in mediating leukemia cell-stroma interactions, multiple antagonists targeting CXCR4 have been developed for use in leukemia treatments^15^,^16^. For example, a small chemical molecule competitive antagonist of CXCR4, AMD3100, was reported for enhanced effect of cytarabine, decreased tumor burden and improved overall survival of AML mice through mobilizing leukemia cells from bone marrow into the peripheral blood^17^. In a phase 1/2 study of relapsed or refractory AML, AMD3100 induced a 2-fold mobilization of leukemic blasts into the peripheral circulation and an overall complete remission of patients when combined with chemotherapeutic drugs mitoxantrone, etoposide or cytarabine^18^. Its analog AMD3465 demonstrated remarkable activity in antagonizing CXCL12-induced CXCR4 signaling pathways, mobilizing AML cells into circulation and enhancing anti-leukemic effects of chemotherapy *in vitro* and *in vivo*^19^,^20^. AMD11070, another derivative of AMD3100, could effectively block stroma-induced migration and adhesion of leukemia cells^21^. When combined with cytotoxic drugs, it could prolong the survival of ALL mice and reduce leukemia cell infiltration into organs. The peptide T140 and its analogs TC14012 and TN14003 derived from horseshoe crabs were reported with effective inhibitory roles mainly in lymphocytic leukemia cells by blocking CXCR4/CXCL12 axis-mediated events and enhancing effect of chemotherapy *in vitro*^16^,^22^,^23^. RCP168 derived from viral macrophage inflammatory protein II is another kind of CXCR4 peptide antagonist that inhibits CXCL12- or MS-5-induced migration of Jurkat or primary CLL cells and increases the drug-induced apoptosis of leukemia cells from patients *in vitro*^20^. However, it is notable that until now, peptide antagonists for AML *in vivo* have been rarely reported. Therefore, it is of great significance to develop novel peptides targeting CXCR4 for providing more therapeutic options in AML treatments.

We have reported a novel peptide E5 by cell-based selection from the *de novo* designed peptides and demonstrated its effect on interfering CXCR4/CXCL12 axis *in vitro*^24^. E5 significantly inhibited migration and adhesion of AML cells to bone marrow stromal cells through inhibiting leukemia cell response to CXCL12- or MS-5-induced activation. Hence we hypothesize that E5 disrupts the interaction of leukemia cells with the bone marrow stromal microenvironment, sensitizing these leukemia cells to toxic drug stresses. In this study, our findings indicate that E5 treatment can diminish marrow stromal protection and confer chemosensitivity to AML cells. By mobilizing leukemia cells from the protective stromal microenvironment, E5 significantly increased the efficiency of chemotherapeutic drugs 10-hydroxy camptothecin, cisplatin or vincristine in *vitro*, leading to an improved overall survival in the mouse model of AML in combination with vincristine or cyclophosphamide.

## Results

### E5 reduces marrow stroma-mediated resistance of leukemia cells to various chemotherapies

**Figure 1:**
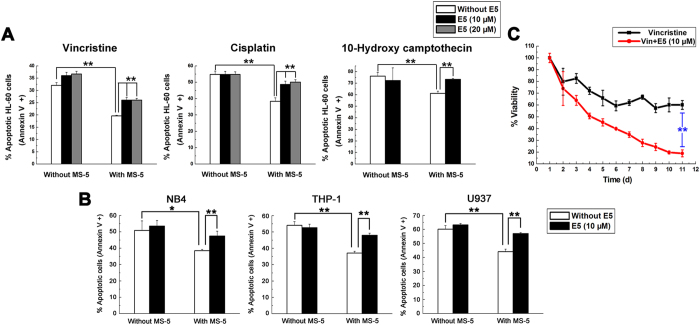
E5 inhibits MS-5 mediated-protection and sensitizes leukemia cells to different chemotherapies. (**A**) E5 enhances vincristine, cisplatin or 10-hydroxy camptothecin-induced apoptosis in HL-60 cells co-cultured with MS-5 cells. HL-60 cells were treated with drugs alone, or drugs in combination with E5 in the absence or presence of MS-5 cells for 48 h. Apoptotic cells were detected by Annexin V flow cytometry. (**B**) E5 enhances 10-hydroxy camptothecin-induced apoptosis in multiple AML cell lines including NB4, THP-1 and U937 cells co-cultured with MS-5 cells for 48 h. (**C**) After continuous combination treatment, vincristine drastically reduces HL-60 cell viability. HL-60 cells growing on MS-5 cell layer were treated with vincristine alone or combined with E5 for 10 days and cell viability was detected using trypan blue every day. Data are presented as mean ± SD (n = 3). The * represents significant difference between two groups (*p < 0.05, **p < 0.01).

In the first set of experiments, we explored whether E5 can overcome stroma-induced drug resistance and sensitize leukemia cells to chemotherapies. The effect of vincristine, cisplatin or 10-hydroxy camptothecin on inducing apoptosis of HL-60 cells co-cultured with the MS-5 cell layer within 48 h was tested with/without E5. It was found that the stromal cell MS-5 dramatically decreased HL-60 cell apoptosis induced by the chemotherapeutic drugs, suggesting protection from drug-induced apoptosis (Fig. 1A), while addition of E5 to the co-culture system removed the protection of MS-5 and sensitized HL-60 cells to the drugs. In the cultivation of the other AML cells (NB4, THP-1, U937), E5 was also found to increase 10-hydroxy camptothecin-induced apoptosis in these cells when MS-5 cells were present (Fig. 1B). It was found that E5 did not enhance the drug-induced apoptosis in culturing systems without MS-5, indicating that the sensitization effect of E5 can be specifically attributed to interfering with the interaction between leukemic cells and stromal cells.

Then we investigated the effect of E5 on 11-day co-culture of leukemia cells and stromal cells. Using trypan blue exclusion method on a daily basis, we observed that when co-cultured with stromal cells HL-60 remained a constant cell viability level of approximate 60.1% after 5 days of treatment with vincristine, suggesting HL-60 became vincristine-resistant (Fig. 1C). Strikingly, when E5 was added to the co-culture system, no vincristine resistance was observed over the whole incubation period. From day 3 the cell viability level of combination group was significantly lower than the group of vincristine alone. After 10 days the cell viability of HL-60 treated in combination of E5 and vincristine was reduced to 19.0%, indicating the drug resistance developed in the co-culture system was overcome by E5 supplement.

### E5-induced leukemia cells mobilization occurs from hematopoietic tissues

**Figure 2:**
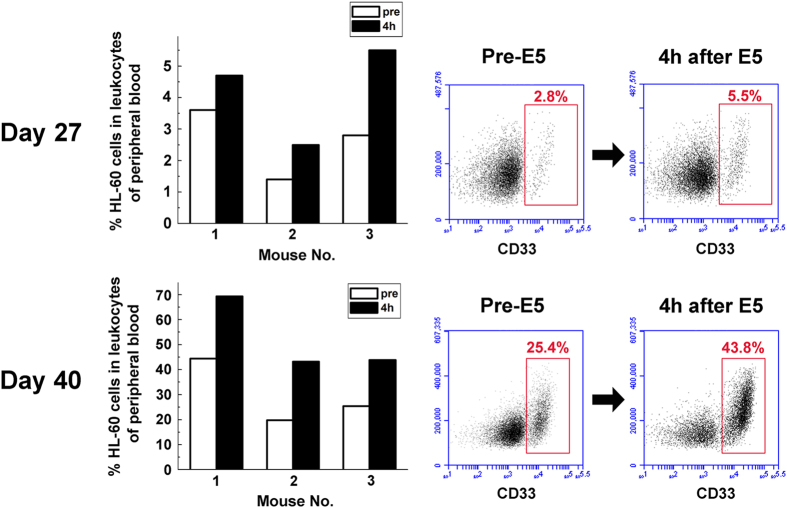
E5 induces a rapid mobilization of leukemia cells into the peripheral blood. Peripheral blood samples were collected through tail vein from three E5-monotreated mice before and 4 h after administration of E5. The percentage of CD33 positive cells (HL-60) was detected with flow cytometry.

We previously reported that E5 can effectively inhibit leukemia cell adhesion to bone marrow stroma and induce leukemia cell detachment from stromal cell layer *in vitro*^24^. Inspired by these results *in vitro*, we translated our findings into an *in vivo* system to confirm its primary mechanism of action. To establish the AML mouse model for treatment, HL-60 cells were injected into sublethally irradiated NOD/SCID mice by tail vein, allowing these cells to migrate to bone marrow and form an enlarged leukemia burden. 20 days after the injection, mice showed marked leukemic symptoms including paralysis in the rear limbs, ruffled fur, and remarkably hunched posture in comparison to healthy control mice. On day 20, 34 and 40 after HL-60 transplantation, cells from bone marrow and spleen of the mice were analyzed with flow cytometry (Supplementary Fig. S1A). The HL-60 cell proportion was 6.1%, 40.4%, 91.9% in bone marrow and 3.3%, 10.2%, 65.2% in spleen, demonstrating the successful establishment of leukemia mouse model (Supplementary Fig. S1B). Then we collected peripheral blood samples from E5-monotreated AML mice before and 4 h after administration of E5 and measured the HL-60 proportion. Results showed that E5 induced a significant increase of circulating HL-60 cells in mice (Fig. 2). Taking one set of flow cytometry data for one of the three mice as an example, the circulating HL-60 proportion increased from 2.8% to 5.5% on day 27 of HL-60 implantation and from 25.4% to 43.8% on day 40. Compared with control, the percentage was almost doubled. These data clearly indicate that E5 mobilizes leukemia cells from stromal microenvironment into peripheral circulation.

### Combination treatment of E5 plus vincristine (Vin) or cyclophosphamide (CTX) prolongs the survival of leukemia mice and reduces leukemia burden

**Figure 3:**
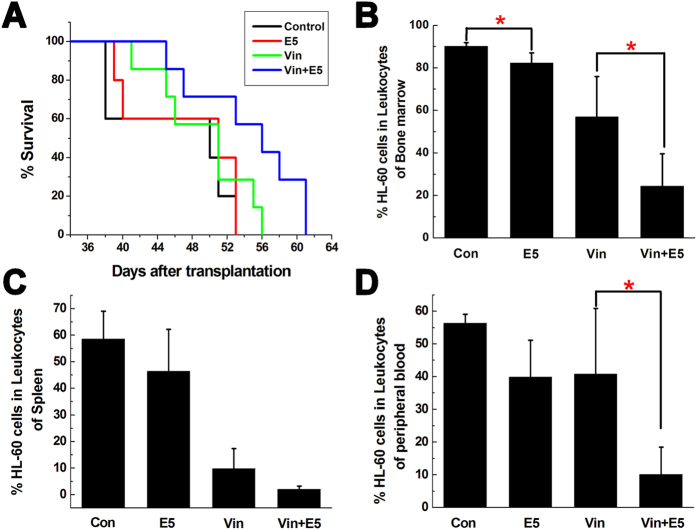
*In vivo* combination treatment of AML mice with vincristine (Vin) and E5. From day 20 of HL-60 transplantation, AML mice received Vin intraperitoneally 4 h after subcutaneous E5 administration twice weekly. (**A**) Overall survival of AML mice treated with sterile water (n = 5), E5 alone (n = 5), Vin alone (n = 7), or a combination of both (n = 7). (**B**–**D**) The percentage of HL-60 cells in the bone marrow, spleen and peripheral blood of AML mice in each group was determined with flow cytometry (n = 4) (*p < 0.05).

**Figure 4:**
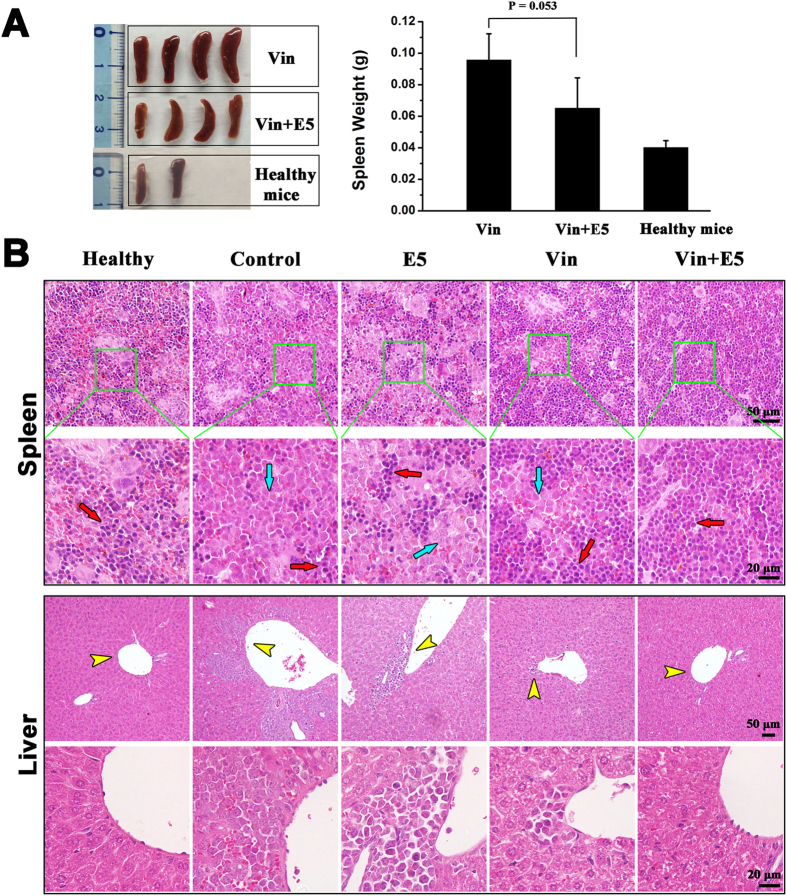
*In vivo* effects of vincristine (Vin) and E5 on infiltration of HL-60 cells into spleen and liver. (**A**) The weight and size of spleens in healthy mice (without HL-60 transplantation) and Vin-monotreated or combination treated AML mice. (**B**) Histologic sections of spleen and liver in each group of mice were stained with H&E. Images of each organ in the second line are the amplification of images in the first line. Red arrows represent normal cells and blue arrows represent HL-60 cells infiltrated in spleens. Yellow arrows represent HL-60 cells infiltrated into livers.

In view of the above results, we combined E5 with chemotherapeutic drugs to examine the elimination effect to the leukemia cells escaped from the protective stromal microenvironment. To ensure that the leukemic cells pushed into the circulation come in contact with the cytotoxic drug, we injected Vin at 4 h after the subcutaneous administration of E5 from day 20 of the HL-60 transplantation. The group receiving combination treatment showed prolonged median survival (56 days) comparing to the group receiving Vin alone (51 days) within the experimental period (*p* = 0.063, Fig. 3A). Analysis with bone marrow, spleen and peripheral blood showed a clear reduction of AML cells load in the combination treatment group compared with that in the Vin-alone treated group (Fig. 3B–D). E5 alone also slightly decreased the HL-60 percentage in bone marrow, spleen and peripheral blood compared with the control group. Besides bone marrow, leukemia cells tend to infiltrate into spleen and liver. Especially spleens, as the largest lymphoid organ, are usually enlarged in the development of leukemia. As shown in Fig. 4A, the spleens of the combination treatment group were markedly smaller than that of the Vin group. From the representative tissue samples for histopathological analysis (Fig. 4B), reticular cells were regularly distributed in spleen of healthy NOD/SCID mice (the mice without HL-60 transplantation). After transplanted with HL-60 cells, splenic tissues were mainly filled with HL-60 cells and the structure integrity was damaged. In the combination treatment group, less HL-60 cell dissemination was found in the spleen and the histologic structure was basically intact compared with the Vin monotherapy group. In addition, for the histopathological analysis of liver, no obvious infiltration of HL-60 cells around blood vessels was visible in the combination treatment group compared with the marked infiltration in the control, E5 and Vin mono-treatment group. These results clearly indicate that combination therapy with E5 and Vin decreased the AML burden in bone marrow, spleen and liver, and induced leukemia remission.

**Figure 5:**
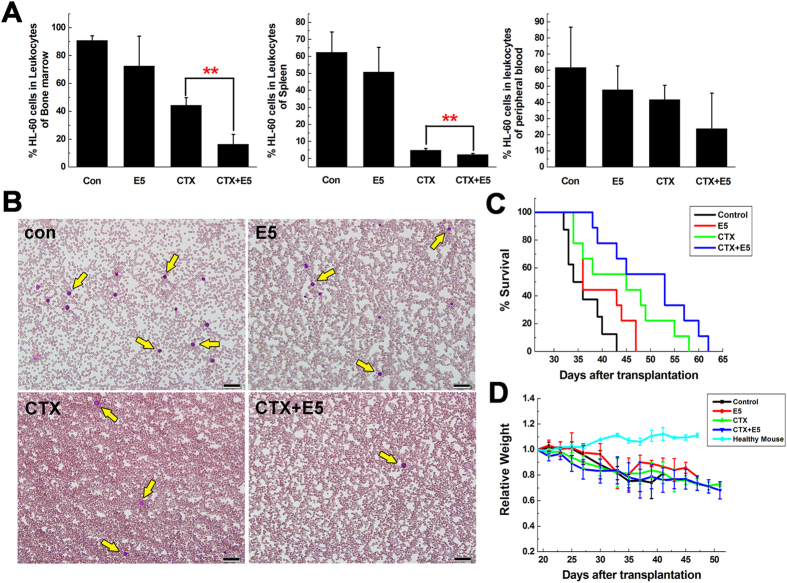
*In vivo* combination treatment of AML mice with cyclophosphamide (CTX) and E5. (**A**) The percentage of CD33 positive cells (HL-60) in the bone marrow, spleen and peripheral blood of AML mice in each group was determined with flow cytometry. Data are presented as mean ± SD (n = 4). The * represents significant difference between two groups (*p < 0.05, **p < 0.01). (**B**) Wright-stained peripheral blood smears of AML mice in each group. Yellow arrows represent HL-60 cells. Scale bar represents 50 μm. (**C**) Overall survival of AML mice treated with sterile water, E5 alone, CTX alone, or a combination of both (n = 9). (**D**) Relative weight of the mice during the period of treatment.

**Figure 6:**
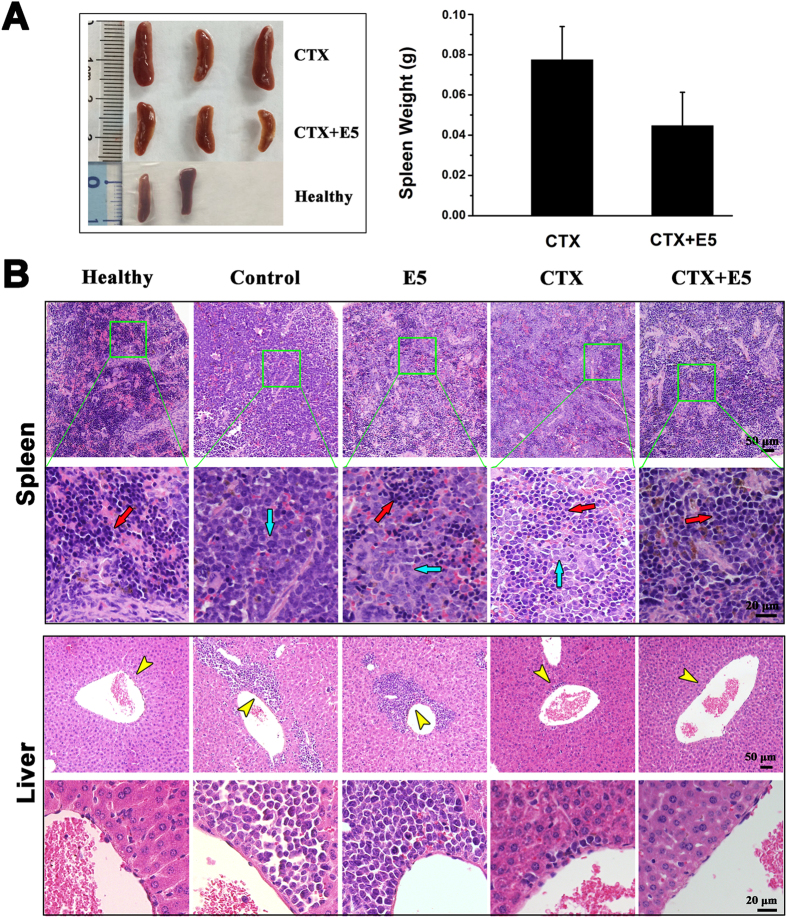
*In vivo* effects of cyclophosphamide (CTX) and E5 on infiltration of HL-60 cells into spleen and liver. (**A**) The weight and size of spleens in healthy mice (without HL-60 transplantation) and CTX-monotreated or combination treated AML mice. (**B**) Histologic sections of spleen and liver in each group of mice were stained with H&E. Images of each organ in the second line are the amplification of images in the first line. Red arrows represent normal cells and blue arrows represent HL-60 cells infiltrated in spleens. Yellow arrows represent HL-60 cells infiltrated into livers.

The effect of E5 plus CTX was also tested in the AML mice model. CTX was injected 4 h after the subcutaneous administration of E5 from day 20 after transplantation. Terminal bone marrow, spleen and peripheral blood samples of all AML mice were analyzed for HL-60 cell proportion with flow cytometry. In the combination treatment group, the percentage of HL-60 cells in the leukocytes collected from bone marrow, spleen and peripheral blood respectively was much lower than that of CTX monotherapy group (Fig. 5A). From the peripheral blood smears with Wright’s staining, a clearly reduced amount of HL-60 cells in combination treatment group (Fig. 5B) was also observed. Meanwhile no hyperleukocytosis or leukostasis was observed after long-term E5 treatment. Ultimately a prolonged median survival was observed in those mice that received the combination treatment (53 days) compared with CTX monotherapy (45 days) (*p* = 0.12, Fig. 5C). E5 alone also slightly extended the survival of leukemia mice. There was no significant effect of drug treatments on the body weights of the mice (Fig. 5D). Moreover, the spleens of the combination treatment group were smaller than that of the CTX group (Fig. 6A). As shown in Fig. 6B, much stronger rescue effect for HL-60 cell dissemination and histologic structural integrity in spleen was observed in combination treatment group. In addition, for the histopathological analysis of liver, no obvious infiltration of HL-60 cells around blood vessels was visible in the combination treatment group compared with the marked infiltration in the control, E5 and CTX mono-treatment group (Fig. 6B). These results clearly indicate E5 treatment significantly enhanced the efficacy of the CTX chemotherapy.

### Systemic toxicity of E5 in healthy mice

We have tested the cytotoxicity of E5 on two nonmalignant cell lines (human umbilical vein cell ea.hy926 and murine stromal cell MS-5) with low level of CXCR4, and confirmed that E5 did not induce cell apoptosis at concentrations ranging from 1 to 80 μM^24^. To evaluate systemic toxicity of E5, we gave healthy BALB/c mice a subcutaneous injection of E5 at 40 mg/kg every other day in one month. After the mice were sacrificed, several organs were examined microscopically. Supplementary Fig. S2A shows representative H&E staining of heart, liver, spleen, lung, kidney, brain and lymph node tissues from mice treated either with sterile water or E5 injection. In particular, no central necrosis was observed in heart, liver, spleen, lung, brain or lymph node, and no tubular necrosis in kidney. There was also no significant effect of E5 administration on the organ weights of the mice (Supplementary Fig. S2B). Meanwhile, routine clinical parameters of serum including alanine aminotransferase (ALT), aspartate aminotransferase (AST), creatinine (Cr(E)) and urea suggest the hepatic and renal function of mice were normal after the long-term E5 treatment (Supplementary Fig. S2C). All these above results demonstrate that there was no damage in these organs of the E5-treated mice and E5 has good security *in vivo*.

## Discussion

Despite significant improvements in the treatment and steady increase in the survival of other types of leukemia, the 5-year survival of patients with AML remains dissatisfactory and a great number of AML new cases and deaths emerge^25^. It has been reported that CXCR4 expression is associated with poor prognosis in patients with AML^26^,^27^. CXCR4 thus can be incorporated into the risk assessment of AML patients^28^. Bone marrow is one of the major tissues that produce CXCL12 which activates CXCR4^29^. Through CXCR4/CXCL12 axis, bone marrow stroma creates a protective microenvironment, providing pro-survival and chemotherapy resistance signals for the homing AML cells^2^. Hence CXCR4 antagonists were developed for leukemia therapy^30^.

Among developed CXCR4 antagonists, peptides are one attractive kind to block CXCR4/CXCL12 axis and abrogate the stromal cell-mediated leukemia cell drug-resistance. In this work, we demonstrated the strong mobilizing activity of E5 using a mouse model of human AML cells. There is a 1.84-fold increase of circulating HL-60 cells in the peripheral blood of AML mice at 4 h after E5 administration, indicating translocation of leukemia cells by E5 from a protective niche to a location where they are more sensitive to therapeutic drug treatment. The results of combination treatment of cytotoxic drugs and E5 were quite remarkable, leading to reduced leukemia burden and prolonged survival. These results still give strong evidence that E5 plays the mobilizing role in the chemotherapies, and we believe the *p* value can be improved by optimizing the leukemia animal model and the overall number of animal. It is also noticeable that very similar treatment effects were obtained no matter using E5 plus Vin or plus CTX, though the mode of action and cellular target of CTX are very different from that of Vin. Hence E5 can be expected to combine with more other cytotoxic drugs in the treatment of leukemia. Another point that should be emphasized is that AML is a heterogeneous disease and can be divided into 8 subtypes according to eight major subtypes from M0 to M7 according to its maturation degree in the French-American-British (FAB) classification system^31^. In the current study, HL-60 and NB4 are defined as subtype M3, THP-1 is defined as M5, and U937 is M6. The experimental results *in vitro* clearly showed E5 can be effective to the different subtypes of AML. For future studies, it is genuinely very interesting to investigate the effects of E5 obtained from HL-60/NOD-SCID model on the other subtypes of AML primary leukemia cells from patients.

Although various CXCR4 antagonists have the function of mobilizing leukemia cells from bone marrow, E5 has one distinguished advantage over others-good safety. Chemical molecule antagonists such as AMD3100 and AMD11070 mobilize leukemia cells into circulation and improve chemotherapy *in vitro* and *in vivo*^17^,^21^,^32^,^33^,^34^. However, AMD3100 is reported to display a weak partial agonist activity^35^,^36^, inducing CXCL12-like G-protein activation in CXCR4-expression cells upon binding of it, which could be a disadvantage to the treatment of leukemia, because CXCR4 activation provides a survival signal for leukemia cells. Long-term animal studies of AMD11070 revealed histological changes in the liver^37^ and a phase 1/2 study suggested its potential toxicity in the liver^38^. Hence, AMD3100 can only be utilized for short-term clinical treatment^39^. In the previous study^24^, we have demonstrated E5 induced neither the CXCR4 activation in four AML cell lines nor apoptosis of two nonmalignant cells (ea.hy926 and MS-5) even at a high concentration. In this work, we show that the long-term E5 treatment didn’t affect the major organ functions and no histological changes were observed, demonstrating the safety of E5. Considering that E5 induced mobilization of leukemia cells out of protection into circulation and enhanced chemotherapy sensitivity, this appears to be a remarkably effective method of boosting the cytotoxicity of low-dose chemotherapeutic drugs on leukemia cells, decreasing the risk of side effects caused by high-dose chemotherapies in leukemia treatments^40^,^41^,^42^. Another advantage of E5 is the low cost. Most of currently reported peptide antagonists are derived from cells by bioengineering technology^15^,^43^,^44^. For examples, T140 and its analogs TC14012 and TN14003 are derived from naturally occurring peptides in horseshoe crabs, and RCP168 is derived from viral macrophage inflammatory protein II. Furthermore, concerns of cytotoxicity have been raised for T140 due to its total positive charge^45^. In contrast, E5 is chemically synthesized according to the sequence of human CXCR4, which makes it more easily, flexibly and economically to be produced.

It should be noted that the relapse of most leukemia cells post chemotherapy is dependent on their intra-and extra-cellular resistances to chemotherapy. The CXCR4/CXCL12 axis mediated drug-resistance of AML cells belongs to the latter. In the current study, the CXCR4/CXCL12 axis is interfered by E5 and lead to the homing AML cells released from bone marrow to blood circulation. As the result, AML cells can be exposed to a higher drug concentration than bone marrow. Nevertheless, intra-cellular drug resistance could be induced for leukemia cells long-term exposed to the drug by expressing extensively drug resistance proteins such as the ATP-binding cassette (ABC) transporter proteins^46^. ABC proteins that confer drug resistance include P-glycoprotein (genes symbol *ABCB1*), the multidrug resistance protein 1 (MRP1, gene symbol *ABCC1*), MRP2 (gene symbol *ABCC2*) and the breast cancer resistance protein (BCRP, gene symbol *ABCG2*), induce the drug efflux from tumor cells and decrease cellular drug accumulation^47^. Several molecules have been developed to inhibit the ABC protein-mediated drug efflux, such as naringenin^48^, pegylated phosphotidylethanolamine^49^, apatinib^50^. The combination therapy of E5 and the ABC protein inhibitors with chemotherapeutic drugs can be expected to overcome the leukemia drug-resistance synergistically.

CXCR4 is also highly expressed by many other malignant cell types, such as breast cancer^51^, lung cancer^52^,^53^, melanoma^54^, prostate cancer^55^, and ovarian cancer^56^, and closely associated with the metastasis, invasion and chemotherapy resistance of these solid tumors. Hence, broader uses of E5 can also be expected in those cancer therapies in combination with chemotherapies.

In conclusion, we here present the evidence that E5 can reduce tumor burden, significantly improve the efficiency of chemotherapies for AML and extend survival in AML mice.

## Materials and Methods

### Cell lines and maintenance

The acute promyelocytic leukemia cell HL-60 and NB4, acute monocytic leukemia cell THP-1, and myelomonocytic leukemia cell U937 were purchased from the Cell Resource Center of Chinese Academy of Medical Sciences (Beijing, China). Murine stromal cell line MS-5 was kindly provided by Professor Bin Yin, the Cyrus Tang Hematology Center, Soochow University, China. Those cells were cultured in 5% CO_2_ at 37 °C in RPMI 1640 (Hyclone Thermo Scientific) supplemented with phenol red, 10% fetal bovine serum (FBS; Gibco, Grand Island, NY), 100 U/mL penicillin, 100 U/mL streptomycin.

### Peptide and antibody

The peptide E5 (GGRSFFLLRRIQGCRFRNTVDD) used in this study was identified by the cell-based selection^24^ and purchased from GL Biochem (Shanghai) Ltd. The stock solution of E5 was dissolved in the sterile water. PE anti-human CD33 antibody was purchased from BioLegend (San Diego, CA).

### *In vitro* combination treatments of E5 with chemotherapeutic drugs

#### Drug combination in short-term

MS-5 cells of 1.5 × 10^5^/well were seeded in 24-well plates in RPMI 1640 containing 10% FBS and incubated overnight to form a stromal cell layer. Leukemia cells of 2.5 × 10^5^ (HL-60, NB4, THP-1 and U937) were treated with E5 at 10 μM or 20 μM in serum-free medium (opti-MEM; Life Technologies, Grand Island, NY) for 1 h, and then cultivated in the presence or absence of MS-5 cell layer for 4 h. After that, chemotherapeutic drugs were added to the culture system (10-hydroxy camptothecin at 40 nM for HL-60, THP-1 and U937, or at 11 nM for NB4; vincristine (Vin; Shenzhen Main Luck Pharmaceuticals, Shenzhen, China) at 2.5 nM and cisplatin (Hospira Australia Pty Ltd, Melbourne, Australia) at 1.2 μM for HL-60) and the cells were cultivated for 48 h. The leukemia cells were then separated from the MS-5 cell layer, washed and stained with FITC-Annexin V (eBioscience, Vienna, Austria) and subjected to C6 Accuri® flow cytometer (Accuri Cytometers, Ann Arbor, MI). In the measurement, 1.5 × 10^4^ cells were collected and the acquired data were analyzed by CFlow Plus software.

### Drug combination in long-term

MS-5 cells of 5 × 10^3^/well were seeded in 96-well plates in RPMI 1640 containing 10% FBS and incubated overnight to form a stromal cell layer. HL-60 cells (2 × 10^4^) were treated with E5 (10 μM) in opti-MEM for 1 h, and then inoculated to the MS-5 cell layer. Either vincristine (1 nM) or a combination of vincristine and E5 (10 μM) was added to the culture system. Half of medium was changed every alternate day and replenished with fresh drug. Care was taken not to destroy the culture system. Viability of HL-60 cells was analyzed by the trypan blue exclusion method every day.

### Establishment of mouse model for human AML

Five-week old female NOD/SCID mice were maintained in the Experimental Animal Center at the Institute of Basic Medical Sciences, Chinese Academy of Medical Sciences (Beijing, China) under specific pathogen-free conditions. All the animal experiments reported herein were carried out in accordance with the approved guideline and approved by the committee on the Animal Care and Use of Institute of Basic Medical Sciences, Chinese Academy of Medical Sciences & Peking Union Medical College. Animals were acclimatized to laboratory conditions for 1 week prior to experiments. HL-60 cells (1 × 10^6^) suspended in 100 μL EDTA/PBS were injected intravenously into the sublethally irradiated (250 cGy) NOD/SCID mice. At about 20 days after transplantation, mice showed leukemia signs. Cells of bone marrow and spleen were collected from the mice and analyzed for HL-60 proportion with flow cytometry on day 20, 34 and 40 after transplantation.

### Treatment in leukemia mouse model with E5 and chemotherapeutic drugs

From day 20, the mice were randomly treated with E5 (10 mg/kg), Vin (0.2 mg/kg), cyclophosphamide (CTX, 90 mg/kg) or a combination of E5 and one of the drugs twice a week. For the combination treatment group, Vin or CTX was injected intraperitoneally 4 h after subcutaneous injection of E5. Mice were monitored for the condition and weight loss. On day 40 after transplantation, mice were sacrificed. Cells of bone marrow, spleen and peripheral blood were collected from each group of mice and analyzed for HL-60 proportion with flow cytometry.

### Flow cytometry analysis

The bone marrow obtained by femur flushing, spleen and peripheral blood from each group of treated mice were collected to prepare single cell suspension. Erythrocytes were excluded using the blood cells lysis buffer (Beckman Coulter, Krefeld, Germany). The human specific CD33 surface marker was used to identify HL-60 (positive rate was 100% as determined by flow cytometry) with PE anti-human CD33 antibody and 1.5 × 10^4^ cells were analyzed with flow cytometry.

### Histological analysis

Livers and spleens from each group of treated mice were fixed in 4% formalin, dehydrated and embedded in paraffin. Tissue sections were prepared and stained with hematoxylin and eosin (H&E). The histology slides were analyzed for HL-60 cells dissemination using an Olympus BX53F microscope equipped with a digital camera (Olympus, Tokyo, Japan) (three representative slides for each group were reviewed).

### Wright staining of blood smears

A drop of peripheral blood from each group of treated mice was pulled between two slides at roughly 45° to create a blood film slide which was allowed to air dry. Slides were briefly fixed in methanol prior to staining with Wright’s stain. Slides were then cover slipped prior to scanning using an Olympus BX53F microscope.

### *In vivo* toxicity of E5

Safety experiments were performed on five-week old healthy female BALB/c mice with four mice per group. The mice were randomly divided into two groups and respectively given a subcutaneous injection of E5 at 40 mg/kg (E5-treated group) or sterile water (control group) every other day. After the 30-day treatment, the mice were sacrificed. Their heart, liver, spleen, lung, kidney, brain and lymph node were weighed and the tissue sections were subjected to H&E staining. Blood samples of each group were collected and centrifuged at 3000 rpm for 15 min at 4 °C to obtain serum. The serum samples were used for routine clinical parameters analysis including alanine aminotransferase (ALT), aspartate aminotransferase (AST), creatinine (Cr(E)) and urea.

### Statistical analysis

All experiments were carried out in triplicate and unpaired Student’s t-test was performed to assess statistical significance of the results (*P < 0.05 and **P < 0.01). The pairwise comparisons for the median survival of leukemia mice between Vin (or CTX) alone group and Vin (or CTX) plus E5 were assessed by using the Log-rank test (SAS 8.2 Software, SAS Institute).

## Additional Information

**How to cite this article**: Li, X. *et al.* Improving chemotherapeutic efficiency in acute myeloid leukemia treatments by chemically synthesized peptide interfering with CXCR4/CXCL12 axis. *Sci. Rep.* **5**, 16228; doi: 10.1038/srep16228 (2015).

## Supplementary Material

Supplementary Data
