# Supplementary material for: Improving chemotherapeutic efficiency in acute myeloid leukemia treatments by chemically synthesized peptide interfering with CXCR4/CXCL12 axis
Source: Sci Rep. 2015 Nov 5;5:16228. doi: 10.1038/srep16228 (PMC4633653; doi:10.1038/srep16228)
Supplement: Supplementary Data [file srep16228-s1.pdf]

Improving chemotherapeutic efficiency in acute myeloid leukemia treatments by chemically synthesized peptide interfering with CXCR4/CXCL12 axis

Xiaojin Li[1*], Hua Guo[1*], Hongyang Duan[2], Yanlian Yang[2], Jie Meng[1], Jian Liu[1], Chen Wang[2¶], Haiyan Xu[1¶]

1. Institute of Basic Medical Sciences, Chinese Academy of Medical Sciences & Peking Union Medical College，Beijing 100005，P. R. China.
2. National Center for Nanoscience and Technology, Beijing 100190, P. R. China

[*] These authors contributed equally to this work.

[¶]Corresponding authors: Haiyan Xu (email: xuhy@pumc.edu.cn; phone: 86-010-69156437; fax: 86-010-69156437) and Chen Wang (email: wangch@nanoctr.cn; phone: 86-010-82545561; fax: 86-010-82545561).

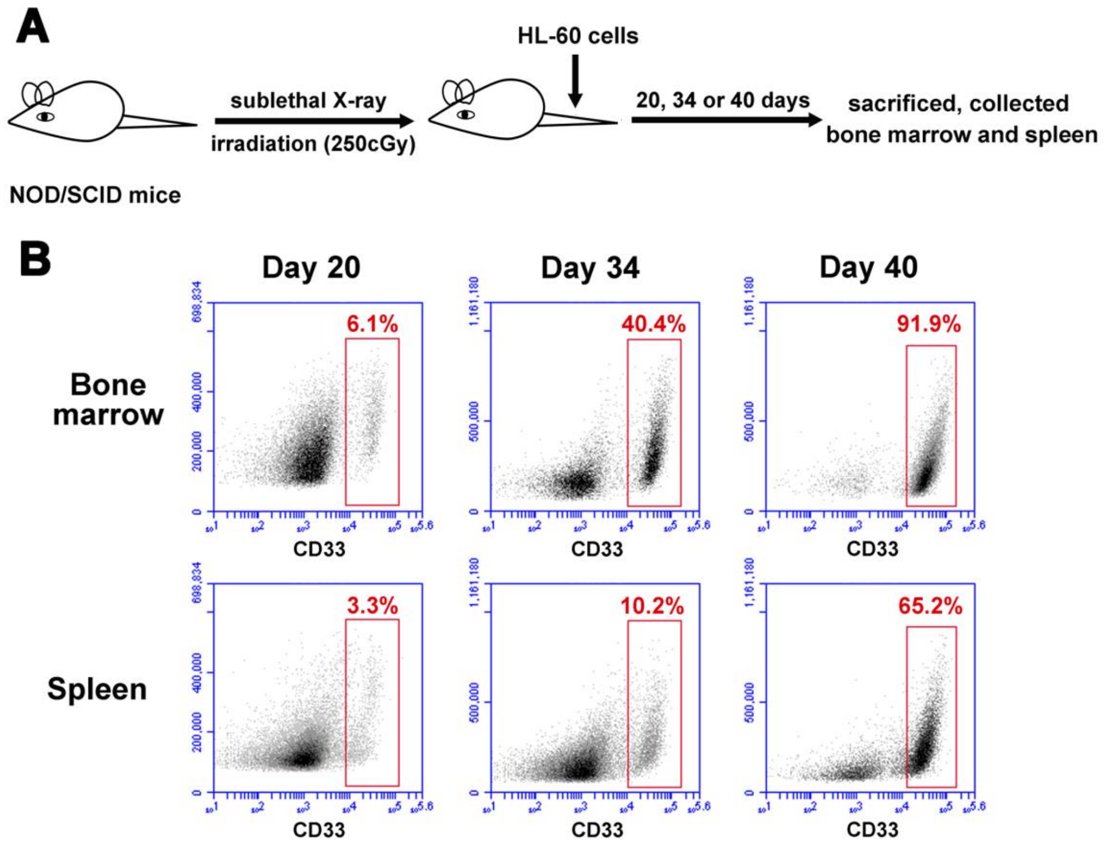

Supplementary Figure S1.

Establishment of AML mouse model. (A) Diagram illustrates the model construction in NOD/SCID mice. After being irradiated by 250 cGy X-ray, mice were intravenously injected HL-60 cells. (B) The proportion of HL-60 cells in bone marrow and spleen of leukemia mice on day 20, 34 and 40 after transplantation. CD33 positive cells were detected using flow cytometry.

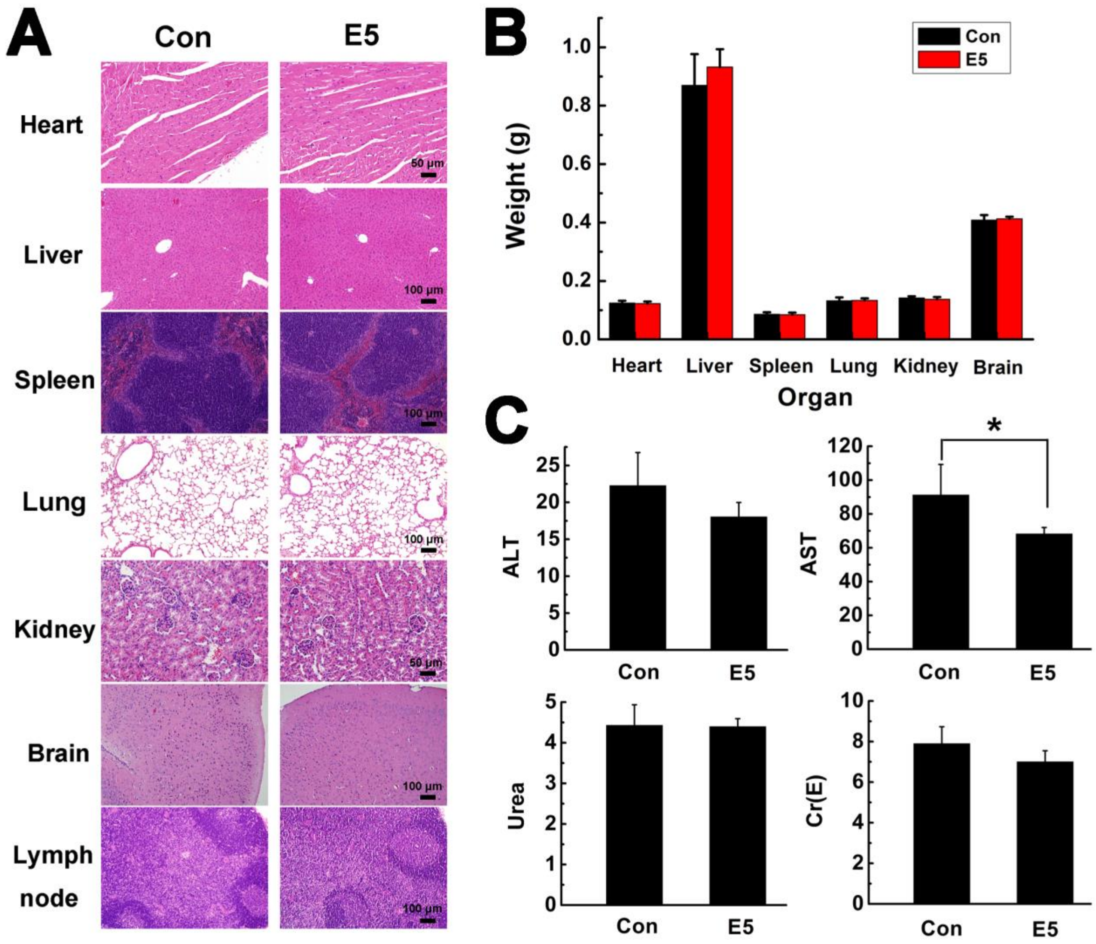

Supplementary Figure S2.

The toxicity evaluation of E5 *in vivo*. The healthy BALB/c mice were given a subcutaneous injection of E5 (40 mg/kg) every other day in one month (n=4). (A) Representative histologic sections of heart, liver, spleen, lung, kidney, brain, lymph node stained with H&E in sterile water (Con) or E5 treated mice. (B) The weight of organs in each group of treated mice. (C) Clinical parameters of serum in each group of treated mice, including alanine aminotransferase (ALT), aspartate aminotransferase (AST), creatinine (Cr(E)) and urea. Data are presented as mean ± SD (*:p<0.05).
